# Characterization of glioma stem-like cells from human glioblastomas

**DOI:** 10.3892/ijo.2015.2992

**Published:** 2015-05-07

**Authors:** SHUN YAMAMURO, YUTAKA OKAMOTO, EMIKO SANO, YUSHI OCHIAI, AKIYOSHI OGINO, TAKASHI OHTA, HIROYUKI HARA, TAKUYA UEDA, TOMOHIRO NAKAYAMA, ATSUO YOSHINO, YOICHI KATAYAMA

**Affiliations:** 1 Department of Neurological Surgery, Nihon University School of Medicine, Tokyo, Japan; 2 Department of Functional Morphology, Nihon University School of Medicine, Tokyo, Japan; 3 Division of Companion Diagnostics, Department of Pathology of Microbiology, Nihon University School of Medicine, Tokyo, Japan; 4 New Energy and Industrial Technology Development Organization, Kanagawa, Japan; 5 Department of Medical Genome Sciences, Graduate School of Frontier Science, The University of Tokyo, Chiba, Japan

**Keywords:** malignant glioma, brain-tumor stem cells, glioma stem-like cells

## Abstract

Glioma stem-like cells (GSCs) could have potential for tumorigenesis, treatment resistance, and tumor recurrence (GSC hypothesis). However, the mechanisms underlying such potential has remained elusive and few ultrastructural features of the cells have been reported in detail. We therefore undertook observations of the antigenic characteristics and ultrastructural features of GSCs isolated from human glioblastomas. Tumor spheres formed by variable numbers of cells, exhibiting a variable appearance in both their size and shape, were frequently seen in GSCs expressing the stem cell surface markers CD133 and CD15. Increased cell nucleus atypia, mitochondria, rough endoplasmic reticulum, coated vesicles, and microvilli, were noted in the GSCs. Furthermore, cells at division phases and different phases of the apoptotic process were occasionally observed. These findings could imply that GSCs have certain relations with human neural stem cells (NSCs) but are primitively different from undifferentiated NSCs. The data may provide support for the GSC hypothesis, and also facilitate the establishment of future glioblastoma treatments targeting GSCs.

## Introduction

Malignant gliomas, especially glioblastomas, represent the most frequent and most lethal primary tumors of the central nervous system. The median survival time and overall survival rate at 5 years of patients with glioblastomas are 14.6 months and only 10%, respectively, despite multimodality treatments including extensive tumor resection, radiotherapy, and chemotherapy (1,2). On the other hand, some cancers have been reported to harbor small cell populations possessing growth sustaining and tumorigenetic abilities. Such cells, termed cancer stem cells (CSCs) or cancer initiating cells, have been identified in certain kinds of tumors including gliomas (2,3). Glioma stem-like cells (GSCs or glioma initiating cells) maintain some properties of cancer stem cells, express genes characteristic of neural stem cells and differentiate into phenotypically diverse populations, including neuronal, astrocytic, and oligodendroglial cells (2,4), and have been reported to contribute to the radioresistance and chemoresistance of gliomas (5,6). GSCs may thus play an important role in tumorigenesis, treatment resistance, and tumor recurrence. Although research on CSCs has consequently received much attention, few ultrastructures of GSCs have, to our knowledge, been adequately described, despite the fact that certain molecular alterations and ultrastructural features of the corresponding tumor cells have been investigated in detail (7,8).

In this study, we provide further data for the biological characterization and morphological description of GSCs using immunocytochemical analysis and transmission electronic microscopy, which may not only provide insight into the oncogenesis of glioblastomas/gliomas but also help in the development of therapies that are suitable for brain CSCs as the target.

## Materials and methods

### Glioma stem-like cells

Two neurosphere-like tumor cell lines (consisting of glioma stem-like cells, glioma initiating cells, brain-tumor stem cells, or glioblastoma stem cells), #0125 and #0222, were provided from Nagoya University School of Medicine, Japan.

The cell lines satisfied the following criteria: a) the cells could be maintained in neurobasal media with N2 (Invitrogen) and B27 supplements (Invitrogen), human recombinant basic fibroblast growth factor (bFGF; R&D Systems), and epidermal growth factor (EGF; R&D Systems) (20 ng/ml each) for 3 months (minimum), and b) 10^3^ of the cells could form tumors in the brain of nonobese diabetic mice with severe combined immunodeficiency disease (NOD/SCID mice), but 10^5^ of the cells after being subcultured with DMEM media (Invitrogen) containing 10% fetal bovine serum could not. These cells were subcultured monthly (minimum) by dissociating the spheres with NeuroCult (StemCell Technologies) (2,9).

### Immunocytochemical staining of GSCs

Immunofluorescence staining for the cancer stem cell markers, CD133 and CD15, was performed to evaluate the characteristics of the GSCs isolated from the human glioblastomas (#0125 and #0222). CD133 is the most accredited marker for CSCs in various organs including glioma, while CD15 is one of the most recently highlighted neural stem cell markers and is also employed to identify CSCs in human brain tumors (3).

Cells were incubated with antibodies against CD133/1 (1:100; 130-080-801, mouse monoclonal IgG1; Milteny Biotec) and CD15 (1:50; MMA, mouse monoclonal; Dako) overnight at 4°C. Appropriate secondary antibodies, anti-mouse immunoglobulins/HRP, Alexa Fluor 594 goat anti-mouse IgM, or Alexa Fluor 488 donkey anti-mouse IgG (following established procedures), were used. Furthermore, for the characterization of GSCs, immunofluorescence was performed with primary antibodies, GFAP for astrocytes (1:100; Millipore: MAB360), Oligo2 for oligodendrocytes (1:100; Product Description: 13999-1-AP), NeuN for neurons (1:100; Millipore: MAB377), and CD34 for endothelial cells (1:100; Immunotech: IM1185). Expression of these cell markers was detected with a laser scanning confocal microscope (Carl Zeiss), and the resultant images were captured on a color CCD at specific magnifications.

### Quantification of MGMT mRNA and protein expression of MGMT on GSCs

Quantitative values for MGMT (O^6^-methylguanine-DNA methyltransferase) mRNA were estimated in GSCs from the human glioblastomas (#0125 and #0222), since the DNA repair enzyme MGMT has been widely considered to be involved in one of the most prominent resistance mechanisms for alkylating anticancer drugs including temozolomide (3-methyl-4-oxo-3,4-dihydroimidazo[5,1-*d*] [1,2,3,5]tetrazine-8-carboxamide; TMZ), a standard therapeutic agent for malignant gliomas (1). The quantification of the *MGMT* gene expression was performed by the real-time quantitative reverse transcription-PCR (RT-PCR) method, as described previously (10).

Furthermore, the expression of MGMT protein in the GSCs (#0125 and #0222) was determined by immunohistochemistry using mouse monoclonal anti-MGMT antibody (MAB16200, clone MT3.1, 1:100; Millipore). Prior to incubation of the primary antibody, a heat-mediated antigen retrieval technique and blocking of endogenous peroxidase activity were carried out. Incubation of the primary antibody was performed for 1 h at 4°C. Diaminobenzidine (DAB) was used for the detection as described previously (11). Nuclei were counterstained with Mayer’s hematoxylin. A negative control was undertaken by omission of the primary antibody.

### Transmission electron microscope examination of the GSC ultrastructure

GSCs from the human glioblastomas were fixed in 1% glutaraldehyde and 0.1 M phosphate buffer for 15 min at 4°C. The cells were washed in phosphate buffer twice for 15 min each. Postfixation was performed in 1% osmium tetroxide for 1 h at 4°C, followed by another two 15-min washes in the same buffer. After dehydration, the material was embedded in Quetol 812 (Nisshin EM) diluted in propylene oxide (1:1) and incubated at room temperature for 24 h. The pellet was then transferred to pure Quetol 812 resin and incubated at 60°C for 72 h, until completely polymerized.

Semithin and ultrathin sections were obtained with the aid of an ultramicrotome. The semithin sections were stained with 1% toluidine blue. The ultrathin sections (100 nm) were placed on copper grids and stained with uranyl acetate and lead citrate. The grids were examined and photographed under a Hitachi H7000 electron microscope.

## Results

### Characterization of GSCs by immunocytochemistry

**Figure 1:**
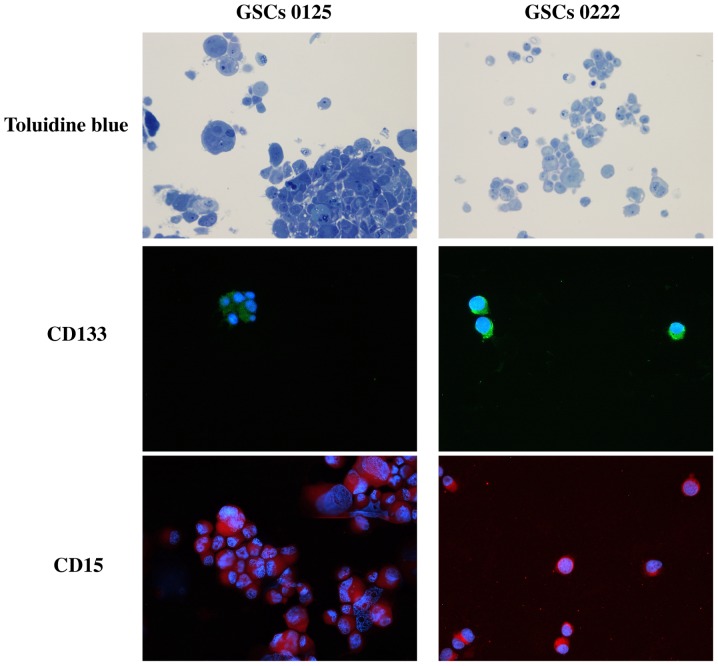
Semithin sections stained with toluidine blue (upper row), glioma stem-like cells (GSCs) from human glioblastoma are gathered together to form tumor spheres. Immunofluorescence staining for CD133 (middle row) and CD15 (lower row), cell membranes of the GSCs are positively stained for CD133 (green) and CD15 (red). Magnifications, ×400. Left, GSCs 0125. Right, GSCs 0222.

Immunofluorescence staining demonstrated that most cells of GSCs 0125 and 0222 expressed the stem cell surface markers CD133 and CD15 (Fig. 1).

**Figure 2:**
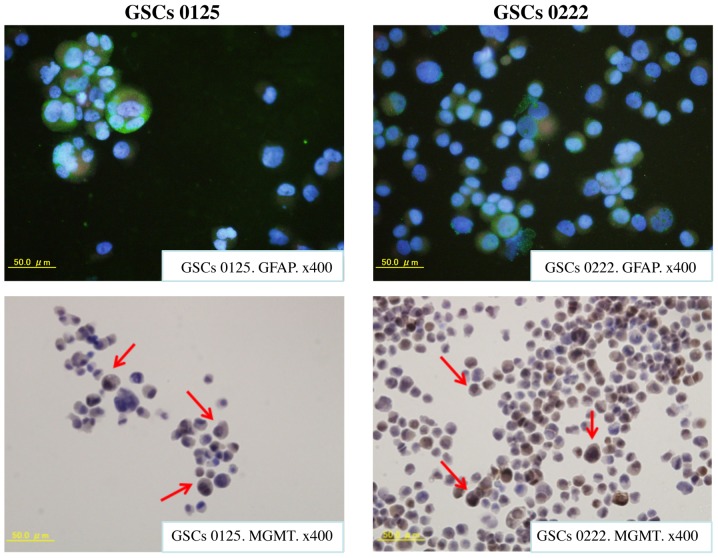
Immunofluorescence staining of glioma stem-like cells (GSCs) for GFAP (upper row), most of the GSCs are positive for the cell marker GFAP. Immunohistological staining for MGMT (lower row), positive expression of MGMT protein is evident in the GSCs. Magnifications, ×400. Left, GSCs 0125. Right, GSCs 0222.

As described above, immunocytochemistry was also performed on GSCs 0125 and 0222 using the following antibodies: GFAP (for astrocytes), Oligo2 (for oligodendrocytes), NeuN (for neurons), and CD34 (for endothelial cells). Most cells of GSCs 0125 and 0222 were stained for GFAP (Fig. 2). However, a few GSCs of 0125 and 0222 were immunopositive for Oligo2, NeuN, and CD34. These experiments demonstrated that the GSCs studied here expressed stem cell markers and differentiated mainly astrocytes.

### Quantitation of MGMT mRNA and protein expression of MGMT on GSCs

The absolute values of *MGMT* mRNA normalized to the level of GAPDH in GSCs 0125 and 0222 were 3.8×10^3^ and 3.1×10^3^, and 5.1×10^3^ and 7.5×10^3^ copies/μg RNA, respectively. These absolute values for *MGMT* mRNA were almost equivalent to those of TMZ-resistant cell lines (10). Furthermore, high expression of MGMT protein was detected in the cell nuclei and cytoplasm of both GSCs 0125 and 0222 (Fig. 2). These findings suggest that the resistance of these cells to alkylating anticancer drugs including TMZ (data for the resistance of these cells to alkylating drugs are not shown here) is probably related to MGMT expression.

### Characterization of GSCs by light and electron microscopy

We employed light microscopy and transmission electron microscopy to observe the morphology of the GSCs. There were no large structural differences between GSCs 0222 and 0125.

**Figure 3:**
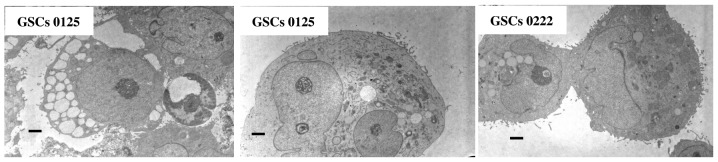
Ultrastructure of GSCs. The cell nuclei of the GSCs are irregular in shape, and the nuclear-cytoplasmic ratio is generally from high to moderate degree. Cellular organelles are generally abundant in the form of mitochondria, rough endoplasmic reticulum, and some vesicles can be seen. Apoptotic cells can be observed in the left image. Bars, 2 μm.

Neurosphere-like clusters (tumor spheres), formed by variable numbers of cells, were frequently seen in the GSCs, with no typical organization (Figs. 1 and 3). They exhibited a variable appearance in both their size and morphology, but none of the cells we studied demonstrated typical features of neurons, ependymal cells, or vessels. The GSCs had many microvilli (like cell surface extensions) that spread throughout the intercellular space. The nuclear-cytoplasmic ratio was generally from high to moderate degree, and the GSCs sometimes had multiple nuclei that were occasionally cleaved (deep indentations) and irregular in shape. The nucleolus was generally prominent but sometimes obscure. Cellular organelles were generally abundant in the form of mitochondria, rough endoplasmic reticulum, and numerous coated vesicles could be seen. Infrequently, cells at division phases and different phases of the apoptotic process were observed (Fig. 3), but typical autophagosomes could not be detected in either a single GSC or cells within the tumor spheres.

## Discussion

The CSC hypothesis suggests that neoplasms are significantly due to a small fraction of cells with stem cell properties (8). There are five main characteristics of CSCs: i) a self-renewal ability, ii) differentiation potential, iii) high tumorigenicity, iv) drug resistance, and v) radioresistance (8,12,13). The above hypothesis must therefore have crucial implications regarding the process of tumorigenesis and choice of therapeutic targets for improving the survival time of cancer patients including glioblastoma patients (6,14). Furthermore, it may help to explain why the present standard treatment can reduce the tumor but often cannot eradicate it resulting in eventual recurrence (6,15,16).

CD133, which was originally detected in neuroepithelial stem cells of mice, is a cell surface marker expressed on human neural stem cells (NSCs) (3,4,17), hematopoietic stem cells (18), and endothelial progenitor cells (18). It is most frequently used as a representative CSC marker, including in gliomas (14,17). CD133-positive (CD133^+^) CSCs have demonstrated a capacity for unlimited self-renewal, as well as an ability to initiate and drive tumor progression in animal models (6,19), and a close correlation has been observed between the expression of CD133 and chemo-resistance and survival in gliomas (6,20,21). However, recent studies have indicated that there are CD133-negative (CD133^-^) GSCs (22), and that the expression of CD133 may reflect the environmental conditions and stress responses such as hypoxia as well as mitochondrial dysfunction (14,17,22). On the other hand, CD15, trisaccharide 3-fucosyl-N-acetyllactosamine, which is known as stagespecific embryonic antigen 1, is strongly expressed in many types of pluripotent stem cells and NSCs in the adult brain (17,23). Furthermore, CD15 was recently proposed to be a marker of stem-like cells derived from brain tumors (14,17). The tumor spheres studied here indicated the existence of CD133^+^ and CD15^+^ GSCs. The data could imply intrinsic relationships between NSCs and GSCs, and suggested that GSCs might retain some characteristics of NSCs (22,24). On the other hand, most cells studied here were CD15-, CD133-, and GFAP-positive, while a few fractions were Oligo2, NeuN or CD34-positive. These findings may imply that the GSCs in the present study were primitively different as compared to undifferentiated NSCs (25). Mao *et al* have previously suggested that the available data have tended to complicate the issue of the source of GSCs, suggesting that GSCs may also contain different types of stem cells which probably originated from different types of NSCs or progenitors (22). However, since normal tissue stem cells and CSCs could well have some similar properties, further studies should focus on the differences, including the molecular genetics and epigenetics, between CD15^+^ and/or CD133^+^ normal tissue stem cells and CD15^+^ and/or CD133^+^ GSCs. Understanding such differences may help to facilitate elucidation of the tumorigenesis and establishment of useful therapies for glioblastomas.

The actual postoperative standard protocol for the treatment of glioblastomas consists of radiotherapy and concomitant TMZ (1). In addition, MGMT hypermethylation (epigenetical silencing of the promoter and coding regions of the MGMT gene) is considered to be one of the principal mechanisms contributing to the TMZ sensitivity of glioblastomas. MGMT removes alkylating adducts from the O^6^ position of guanine and protects cells from cytotoxic and mutagenic effects, conferring a resistance of the tumor cells to alkylating agent chemotherapy including TMZ (10). More recently, the results of the EORTC-NCIC trial have established a predictive value for *MGMT* methylation status for the benefit from TMZ treatment achieved by patients with glioblastoma (1). Currently available data suggest that resistance of GSCs to chemotherapy may be significantly linked to MGMT (6), which is consistent with the results showing higher *MGMT* mRNA and protein expression of GSCs in the present study.

The observed ultrastructural features were similar in both GSCs 0125 and 0222. The nuclear-cytoplasmic ratio was generally from high to moderate degree, and the GSCs sometimes had multiple nuclei that were occasionally cleaved (deep indentations) and irregular in shape. Cellular organelles were generally abundant in the form of mitochondria, rough endoplasmic reticulum, and numerous coated vesicles could be seen. These ultrastructures also implied that the GSCs were primitively differentiated as compared to undifferentiated NSCs (26). Thus, the ultrastructural features observed in this study were approximately in agreement with those described in previous reports (7,8). Atypia of the cell nucleus was apparent in the GSCs, indicating that the degree of malignancy of these cells including their invasive ability tends to be high (8). Rough endoplasmic reticulum was rich in the cytoplasm of the GSCs. It has been suggested that rough endoplasmic reticulum may play an important role after exposure to radiotherapy/chemotherapy in preferentially activating protein synthesis and repair of cell damage, because it is covered with ribosomes which contribute to mRNA translation into proteins (8). Microvilli were also rich in the presently studied GSCs. Cell surface expression of microvilli may help to protect GSCs from the immune system, cytotoxic effector cells, so that they survive more easily than common/usual glioma cells (8,27,28). Microenvironmental cues and cell-cell interactions in the adult brain participate in the regulation of stem cell quiescence and proliferation, and of the neurogenetic or gliogenetic lineage (17). The microvilli of GSCs are also considered to play an important role in the receipt of signals from the microenvironment and cell-cell interactions related to the maintenance of GSCs.

On the other hand, some EGF-expanded free-floating neurosphere cells derived from rat fetal striatum possess a single cilium typical of early neural precursors (26). Furthermore, it has been demonstrated that ependymal cells are capable of forming spherical clones, and some of the cells within these spheres maintain the ependymal phenotype as indicated by extensive ciliation (30–50 per cell) (26). In the present study, we did not observe a single cilium or multiple cilia in the GSCs. These findings seem reasonable because most of the GSCs in the present study were stained for CD15, which has been described as a marker for NSCs that do not contain ependymal cells (22). Moreover, it has been suggested that ependymal cells or astrocytes may originate from multi-potent adult NSCs (26,29). Such considerations complicate our understanding of the source of GSCs, which probably originate from different types of NSCs or progenitors.

Most normal stem cells are considered to be at G_0_ stages of their cell cycles, but a few GSCs at division phases were observed in the present study, which was consistent with the proliferation capabilities of GSCs. Further, the apoptotic process could be detected, but typical autophagosomes could not be observed in either a single GSC or cells within the tumor spheres. Zhao *et al* have reported that apoptosis bodies and autophagosomes could barely be found in their ultrastructural studies on glioma stem cells/progenitor cells, and presumed that deficiencies of apoptosis may originate in deficiencies of autophagy (7). Kim *et al* (14) and Eramo *et al (*30) suggested that drug resistance observed in GSCs might depend on abnormalities of the cell death pathway, such as overexpression of anti-apoptotic factors or silencing of key death effectors. The discrepancies regarding apoptosis in GSCs thus require further examination. On the other hand, there is growing evidence to support the participation of autophagy in processes such as cellular differentiation (7). It may thus be reasonable that autophagosomes were not found in the GSCs, because the GSCs in the present study mainly contained CD133^+^ and CD15^+^ cells indicating that these cells were immature.

In conclusion, although further confirmative studies need to be undertaken, the antigenic characteristics and ultrastructural features of the GSCs isolated from human glioblastomas, could imply that GSCs have certain relations with NSCs but are primitively different from undifferentiated NSCs. The data may support the GSC hypothesis suggesting an important role in tumorigenesis, treatment resistance, and tumor recurrence, and also facilitate the development of future glioblastoma therapies targeting GSCs.
